# Deciphering Genomes: Genetic Signatures of Plant-Associated *Micromonospora*

**DOI:** 10.3389/fpls.2022.872356

**Published:** 2022-03-25

**Authors:** Raúl Riesco, Maite Ortúzar, José Manuel Fernández-Ábalos, Martha E. Trujillo

**Affiliations:** Department of Microbiology and Genetics, University of Salamanca, Salamanca, Spain

**Keywords:** genome, *Micromonospora*, microbe-plant interaction, endophyte, actinobacteria, PGP

## Abstract

Understanding plant-microbe interactions with the possibility to modulate the plant’s microbiome is essential to design new strategies for a more productive and sustainable agriculture and to maintain natural ecosystems. Therefore, a key question is how to design bacterial consortia that will yield the desired host phenotype. This work was designed to identify the potential genomic features involved in the interaction between *Micromonospora* and known host plants. Seventy-four *Micromonospora* genomes representing diverse environments were used to generate a database of all potentially plant-related genes using a novel bioinformatic pipeline that combined screening for microbial-plant related features and comparison with available plant host proteomes. The strains were recovered in three clusters, highly correlated with several environments: plant-associated, soil/rhizosphere, and marine/mangrove. Irrespective of their isolation source, most strains shared genes coding for commonly screened plant growth promotion features, while differences in plant colonization related traits were observed. When *Arabidopsis thaliana* plants were inoculated with representative *Micromonospora* strains selected from the three environments, significant differences were in found in the corresponding plant phenotypes. Our results indicate that the identified genomic signatures help select those strains with the highest probability to successfully colonize the plant and contribute to its wellbeing. These results also suggest that plant growth promotion markers alone are not good indicators for the selection of beneficial bacteria to improve crop production and the recovery of ecosystems.

## Introduction

The relationship between plants and microbial communities present in the soil is highly complex. These communities and especially those associated with the rhizosphere fluctuate in response to the surrounding environment which is affected by biotic and abiotic parameters (Sun et al., 2021; Yukun et al., 2021; Zhang et al., 2021). The collective communities of plant-associated microorganisms are known as the plant microbiome (Mendes et al., 2013) and play a major role in plant health and adaptation to environmental factors (Yukun et al., 2021).

In shaping the plant microbiome, plants select for those microbial partners that will contribute to improve its growth and resilience. In return, the microbiota associated will be provided with nutrients, mainly secreted as root exudates (Zhang et al., 2021). All together, they establish complex microbial-plant and microbe-microbe interactions (microbial networks) to produce a particular plant phenotype (Reid and Greene, 2012; Toju et al., 2018).

Hitherto, only a few individual effects that plants and microbes have on each other have been well-characterized (e.g., nitrogen fixation of rhizobia). However, it is essential to understand how different interactions combine to produce a particular function (chemical, genetic, and/or physical) in a highly dynamic environment (Reid and Greene, 2012). Levy and colleagues recently reported that plant- and root-associated bacteria contained enriched genomes with significant overlap of the same function (e.g., carbohydrate metabolism) indicating an evolutionary adaptation to a specific niche (Levy et al., 2018) and suggesting a common strategy across diverse bacterial taxa to adapt to a plant environment.

Understanding plant-microbe interactions with the possibility to modulate the plant’s microbiome is essential to design new strategies for a more productive and sustainable agriculture (Finkel et al., 2017; Benito et al., 2022). Most bacterial inoculants currently used to improve crops are composed of a single strain randomly isolated and equipped with a set of traits known as plant growth promotion (PGP) (Bulgarelli et al., 2013; Finkel et al., 2017; de Souza et al., 2019). In addition, many of the PGP features have been determined by *in vitro* screening assays or inoculation experiments under controlled conditions, rarely tested in the field (de Souza et al., 2020). Despite being broadly adopted, these strategies fail to capture important aspects of plant–microbe interactions (de Souza et al., 2019). To improve the use of bioinoculants, synthetic microbial communities are gaining a lot of interest as they can be custom built based on information derived from their ecology and genetics and translated into predictable traits. Thus, a key question is how to design bacterial consortia that will yield the desired host phenotypic outputs (Herrera Paredes et al., 2018). Together with the need to understand how microbial communities interact and shape the plant microbiome, it is also necessary to learn about the function and contribution of individual microorganisms, at the organismal/molecular level, to design manageable and traceable consortia containing all needed functions for a successful interaction (Vorholt et al., 2017).

Bacterial plant colonization is also a crucial step. In a recent study, a set of genomic features for bacteria with high capacity for plant colonization was identified (de Souza et al., 2019). The combination of colonization features and specific functions that confer benefits to the plant growth are, therefore, essential to design bacterial consortia to apply to crops.

It is logical to assume that bacteria closely related to plant/rhizosphere habitats would present a higher potential to interact with a plant and contribute to the host phenotype. It is likely that bacterial communities from a specific niche evolved and present characteristic traits (metabolism, biofilm formation, etc.) not found in individuals from other habitats (e.g., soil, sediments, marine, etc.) (Vorholt et al., 2017; Levy et al., 2018).

*Micromonospora* is a cosmopolitan actinobacterium widely found in diverse environments, especially soil, marine, and freshwater habitats (de Menezes et al., 2012; Genilloud, 2015). In the last decade, many micromonosporae have been reported from diverse plant tissues, specially from nitrogen fixing nodules (Trujillo et al., 2010; Carro et al., 2013; Benito et al., 2022) and this bacterium has been shown to closely interact with plants acting as a helper bacterium (Trujillo et al., 2014; Martínez-Hidalgo et al., 2015). This work was designed to identify the potential genomic features involved in the interaction between *Micromonospora* and known host plants. Seventy-four *Micromonospora* genomes representing diverse environments were used to generate a database of all potentially plant-related genes using a novel bioinformatic pipeline that combined screening for microbial-plant related features and comparison with available plant host proteomes. After this, a comparative genomic analysis based on the newly generated database was performed. Our results indicate that the identified genomic signatures help select those strains with the highest probability to successfully colonize and contribute to the wellbeing of the host plant. This strategy could be useful for the selection of other taxa using appropriate databases. The use of genome sequence data to define genomic signatures would be an excellent alternative to the limiting information obtained from defining PGP features.

## Materials and Methods

### Isolation of Strains, Genome Sequencing, and Phylogenomics

Seventeen *Micromonospora* strains isolated from nodules and leaves of six different legumes, as described before (Trujillo et al., 2010) were selected for whole genome sequencing (Table 1) with Illumina MiSeq. DNA preparation and sequencing followed methods described previously (Riesco et al., 2018). Reads were assembled with SPAdes v. 3.10.1 (Bankevich et al., 2012) and protein coding sequences (CDSs) were predicted using Prodigal v. 2.6.2 (Hyatt et al., 2010). Up to date Bacterial Core Gene (UBCG) tool^1^ was used for phylogenomic tree reconstruction, using codon-based alignment and filtering all gap-containing positions. Visualization of the phylogenomic tree was made using iTOL online viewer (Letunic and Bork, 2021), with the aid of table2itol R script.^2^

**TABLE 1:** Source of strains used in this study and identification according to the 16S rRNA gene sequence.

Strain	Host plant	Isolation	Plant collection site	Geographical coordinates	Identification (16S rRNA)	References
GAR05	*Cicer arietinum*	Nodule	Cabrerizos	40° 58′ 40″ N; 5° 35′ 56″ W	*M. saelicesensis* (99.9%)	Riesco et al., 2018
GAR06	*C. arietinum*	Nodule	Cabrerizos	40° 58′ 40″ N; 5° 35′ 56″ W	*M. saelicesensis* (100%)	Riesco et al., 2018
LAH08	*Lupinus angustifolius*	Leaf	Cabrerizos	40° 58′ 39″ N; 5° 35′ 48″ W	*M. noduli* (99.9%)	Riesco et al., 2018
LAH09	*L. angustifolius*	Leaf	Cabrerizos	40° 58′ 39″ N; 5° 35′ 48″ W	*M. zamorensis* (100%)	This study
Lupac 06	*L. angustifolius*	Nodule	Saelices	40° 40′ 06″ N; 6° 38′ 02″ W	*M. saelicesensis* (99.9%)	Trujillo et al., 2007
Lupac 07	*L. angustifolius*	Nodule	Saelices	40° 40′ 06″ N; 6° 38′ 02″ W	*M. saelicesensis* (99.9%)	Trujillo et al., 2007
MED01	*Medicago* sp.	Nodule	Salamanca	40° 57′ 26″ N; 5° 39′ 37″ W	*M. arida* (99.9%)	This study
MED15	*Medicago* sp.	Nodule	Salamanca	40° 57′ 26″ N; 5° 39′ 37″ W	*M. noduli* (100%)	Riesco et al., 2018
ONO23	*Ononis* sp.	Nodule	Cabrerizos	40° 58′ 40″ N; 5° 35′ 56″ W	*M*. *noduli* (100%)	Riesco et al., 2018
ONO86	*Ononis* sp.	Nodule	Cabrerizos	40° 58′ 40″ N; 5° 35′ 56″ W	*M*. *noduli* (99.9%)	Riesco et al., 2018
GUI43*^T^*	*Pisum sativum*	Nodule	Cañizal	41° 10′ 04″ N; 5° 22′ 08″ W	*M*. *noduli* (100%)	Carro et al., 2016
PSH03	*P. sativum*	Leaf	Salamanca	40° 57′ 24″ N; 5° 39′ 31″ W	*M*. *arida* (99.7%)	This study
PSH25	*P. sativum*	Leaf	Salamanca	40° 57′ 24″ N; 5° 39′ 31″ W	*M. zamorensis* (99.7%)	This study
PSN01	*P. sativum*	Nodule	Salamanca	40° 57′ 24″ N; 5° 39′ 31″ W	*M. saelicesensis* (99.9%)	Riesco et al., 2018
PSN13	*P. sativum*	Nodule	Salamanca	40° 57′ 24″ N; 5° 39′ 31″ W	*M. saelicesensis* (99.9%)	Riesco et al., 2018
NIE111	*Trifolium* sp.	Nodule	Villamanta	40° 17′ 45″ N; 4° 6′ 48″ W	*M. saelicesensis* (99.9%)	This study
NIE79	*Trifolium* sp.	Nodule	Villamanta	40° 17′ 52″ N; 5° 6′ 56″ W	*M. saelicesensis* (99.9%)	This study

### Data Compilation and Proteome Annotation

Fifty-four available *Micromonospora* genomes were retrieved from GenBank and IMG depositories (Markowitz et al., 2012; Clark et al., 2016). Additionally, three *Salinispora* genomes were included given their close phylogenetic relationship with *Micromonospora* and their unique marine obligate lifestyle (Millán-Aguinãga et al., 2017; Carro et al., 2018; Riesco et al., 2018). All genomes were checked for contamination using CheckM in KBase environment (Parks et al., 2015; Arkin et al., 2018).

For data normalization, the 74 bacterial proteomes were re-annotated. HMMER v. 3.1b2 (hmmer.org) was used to annotate all proteomes against Pfam v. 31.0, TIGRFAM v. 15.0 and the *Genomic Features of Bacterial Adaptation to Plants* (GFOBAP) HMM protein profiles (Haft, 2001; Finn et al., 2016; The UniProt Consortium, 2017; Levy et al., 2018). EggNOG-mapper online tool (Huerta-Cepas et al., 2017) was used to annotate all proteomes against the EggNOG v. 4.5.1 bacterial database (Huerta-Cepas et al., 2016).

### Construction of the *Micromonospora* Database

A cut-off BLAST value was calculated using a pre-established bacterial core-gene set comprising 92 bacterial genes described in the UBCG method (Na et al., 2018). All genomes were screened for these markers and aligned using UBCG 3.0 (Na et al., 2018). Identity matrices were calculated for all alignments, and the mean maximum, and minimum percentages were determined. Roary v. 3.11.2 (Page et al., 2015) was used to define the core and pan-genomes, using the previously calculated BLAST identity cut-off for the clustering of proteins.

The selection of plant-related bacterial genes (PR) was based on a pre-defined dataset of plant-associated annotation features included in the GFOBAP database (Levy et al., 2018). Considering the phylogenetic position of *Micromonospora* (Nouioui et al., 2018), the dataset was restricted to the first group of the *Actinobacteria* (*Actinobacteria*1 database). Orthofinder groups, COGs, KEGG Orthologs (KO), Pfam and TIGRFAM within *Actinobacteria*1, “Reproducible Plant Associated Domains” and “Plant-Resembling Plant-Associated and Root-Associated Domains” (PREPARADOs) were used. Annotations of the bacterial genomes were screened against GFOBAP database using *data.table* v. 1.13.6 and *tidyr* v. 1.1.2 packages (Finn et al., 2016; Wickham and Henry, 2018) in R v. 3.6.2 (R Development Core Team and R Core Team, 2011), and only those supported by two or more statistical approaches as described in the original database were considered (Levy et al., 2018).

Proteomes of known *Micromonospora* host plants were screened in UniprotKB database (release 2018_6) (The UniProt Consortium, 2017). Eighteen proteomes, comprising different species of *Cicer, Glycine*, *Lupinus*, *Medicago*, *Oryza*, *Phaseolus*, and *Trifolium* were used to create a BLAST database, comprising 731,325 proteins.

Proteomes of the 74 bacterial strains were blasted against the plant proteome database, using BLASTp included in BLAST + executables v. 2.7.1 (Camacho et al., 2009), with a threshold of 1e^–30^ for the E-value, 70% coverage and 30% identity. All identified coding genes found in the analysis were labeled as “plant-resembling bacterial genes” (PRB).

### *Arabidopsis* Plant Assays

Nine strains randomly selected from clusters 1 (MED15, PSN01, and PSH03), 2 (*M. aurantiaca* DSM 43813*^T^*, *M. chaiyaphumensis* DSM 45246*^T^*, and *M. chalcea* DSM 43026*^T^*), and 3 (*M. pattaloongensis* JCM 12833*^T^*, *M. palomenae* DSM 102131*^T^*, and *M. olivasterospora* DSM 43868*^T^*) were used to inoculate *Arabidopsis thaliana* Col0 seedlings in axenic conditions. Forty plants per strain were prepared and inoculated as described previously (Ortúzar et al., 2020). After 4 weeks, root length, rosette leaves diameter, number of flowers, and fruits were registered. Data were standardized using Z-scores and analyzed by Kruskal-Wallis test. Principal component analysis (PCA) was used to associate the parameters measured with the strains.

### Statistical Analyses and Phylogenomic Reconstruction

Kruskal-Wallis test (*p* < 0.05) was used to determine the relationships between habitat and *Micromonospora* genome lengths; number of potential plant-related genes, and habitat (IBM^®^ SPSS^®^ Statistics v.25). Bar-plots for COG analyses were made using *ggplot2* v. 3.3.3 and *ggfortify* v. 0.4.11 packages (Tang et al., 2016; Wickham, 2016). *FactoMineR* v. 2.4, *factoextra v. 1.0.7*, *FactoInvestigate* v. 1.7, and *cluster* v. 2.1.1 packages (Lê et al., 2008; Kassambara and Mundt, 2017; Maechler et al., 2018; Thuleau and Husson, 2018) were used for PCA and cluster analysis of the COGs and plant-related functional KEGG characterization. KEGG annotations were compared using *factoextra* package for PCA and hierarchical clustering. Unique strain KEGG elements were deleted from the analysis.

**FIGURE 1:**
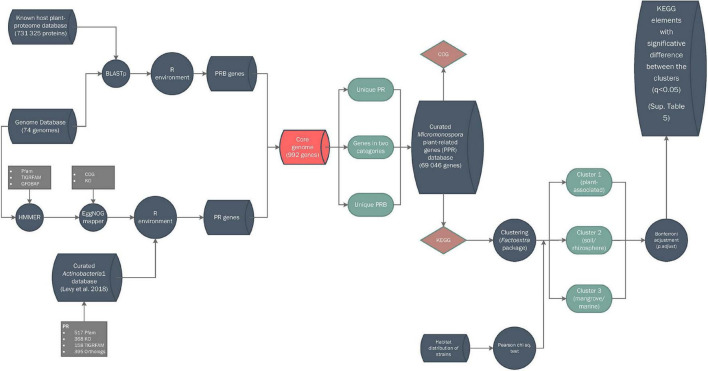
Bioinformatic workflow for database construction. Symbols: cylinders, databases; circles, pipeline processes; rhomboids, annotations; squares, input data; balloons, partial results.

*P*-values (hereafter, *q* value) generated in KEGG abundance analysis were corrected using *p.adjust* tool, included in *stats* R native package, with Bonferroni adjustment method (R Development Core Team and R Core Team, 2011; Jafari and Ansari-Pour, 2019) and q < 0.05 was used for KEGG elements abundance in each calculated cluster, unless stated otherwise. A Pearson’s chi-square test and a contingency table analysis using multiple regression were used to study the clusters generated in the KEGG analysis and the habitat distribution of the strains in each cluster (Beasley and Schumacker, 1995) (IBM^®^ SPSS^®^ Statistics v.25). *ComplexHeatmap* v. 2.2.0 package (Gu et al., 2016) was used for heatmap constructions. A flowchart explaining how the database was constructed is provided in Figure 1.

## Results

### Genomic Features and Habitat Distribution

Genome size ranged from 6.8 to 7.6 Mb (mean 7.1), with isolates PSH25 and MED01 having the smallest and largest genomes, respectively. Other genome characteristics including number of coding DNA, tRNAs, rRNAs, and regularly interspaced short palindromic repeat sequences (CRISPR) are summarized in Supplementary Table 1. The 74 bacterial genomes represented soil/rhizosphere (39%), plant-associated (34%), mangrove/marine sediments (19%), and other environments (8%) (Supplementary Table 2). No correlation was found between the *Micromonospora* genome sizes and their specific habitats (*p* < 0.05) (Supplementary Figure 1). The plant-associated (PA) strains showed similar genome lengths with a mean of 7.1 ± 0.4 Mb. The genomes of the remaining habitats presented higher dispersion values, but their sizes were very similar to the PA strains (soil/rhizosphere, 7.1 Mb ± 0.4; marine/mangrove sediments 6.7 ± 0.7; others 6.8 ± 0.4). PCA of the COG distributions and their relation to the strain habitats were highly influenced by transcription (K, ∼30%), replication and repair (L, ∼26%), carbohydrate metabolism and transport (G, ∼16%), and secondary metabolism (Q, ∼12%) gene categories, accounting for 84% of the variance (Supplementary Figure 2). The PA strains were recovered as a well-recognized cluster highly influenced by the K and G categories, as reported for other plant-related bacteria (Levy et al., 2018; Pinski et al., 2019). On the contrary, the strains representing the remaining habitats were highly dispersed with no apparent correlation. The complete COG distribution of each strain is given in Supplementary Table 3.

### Genomic Features and Functional Diversity of Plant-Related *Micromonospora*

The *Micromonospora* core genome based on an identity threshold of 70% protein homology contained 992 genes (15.5% for an average genome of 6,407 genes). This data was labeled as not differential and removed. In addition, 307 ± 38 genes (per genome) labeled as “plant-resembling genes” in the BLASTp query against the host plant proteomes were included in the gene pool (Supplementary Table 4). The above data, together with the plant-related annotation features supported by two or more statistical analyses derived from the GFOBAP database (517 Pfam, 368 KEGG Orthology (KO), 158 TIGRFAM, and 395 Orthofinder-generated orthologs) were combined for a final database of 69,046 putative plant-related genes (PPR) (Supplementary Table 4).

The distribution of putative plant-related genes varied among strains, with *M. pisi* DSM 45175^T^ showing the highest number (1,137), followed by *M. cremea* DSM 45599^T^ (1,121). As expected, the *Salinispora* strains had the lowest number of PPR genes (570-629). The plant-associated strains showed the highest number of PPR genes as compared to those from other environments (q < 0.01) with a mean of 1036 ± 58 (Supplementary Figure 3).

**FIGURE 2:**
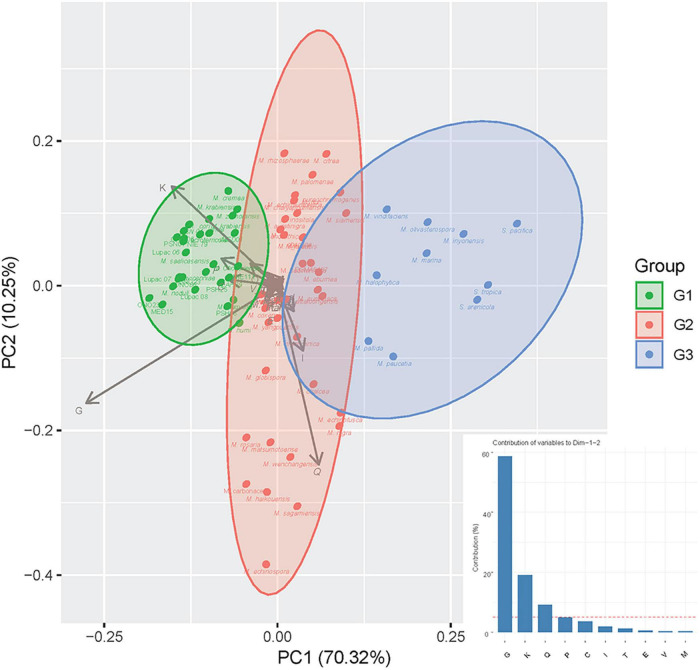
Principal component analysis (PCA) of putative plant-related genes according to COG categories with strains recovered in groups G1, G2, and G3, respectively (for Group composition, see Section “Results”). The bar graph represents the contribution (%) of each COG category to dimensions 1 and 2 of the PCA.

Principal component analysis of the putative plant-related gene COGSs represented in the curated database (69,046) revealed a distribution highly dependent on four categories: carbohydrate metabolism and transport (G, ∼60%), transcription (K, ∼20%), secondary metabolism (Q, ∼10%), and inorganic ion transport and metabolism (P, ∼5%) (Figure 2). Based on the COG annotations, the *Micromonospora* strains formed three groups: the first one (G1), comprised 29 strains of which 22 were plant-associated (76%), six soil/rhizosphere-related (21%), and a single mangrove/marine sediment isolate (3%). This group was highly influenced by K, G, and P categories, showing a compact distribution (Figure 2). Thirty-five strains made up a highly heterogeneous group, G2, 18 from soil/rhizosphere (51%), eight from mangrove/marine sediments (23%), nine plant-associated (3%), and six from other environments (17%); highly impacted by secondary metabolism. Group 3 (G3) contained 10 isolates, five from soil/rhizosphere and 5 from mangrove/marine sediments which included the three *Salinispora* strains. Unlike G1, groups 2 and 3, appeared more scattered, showing the diverse origin of the strains (Figure 2).

**FIGURE 3:**
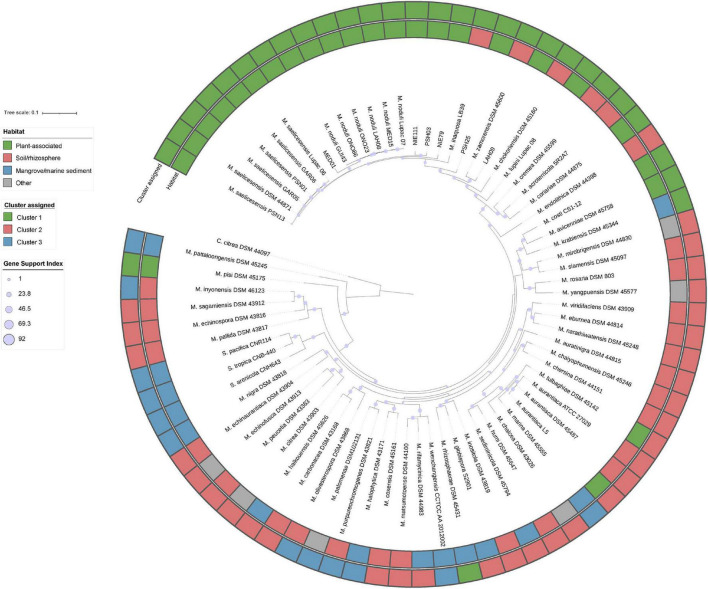
Up-to-date bacterial core gene phylogenomic tree reconstructed with 92 bacterial core genes. Gene support indices are given at nodes as filled purple circles.

KEGG annotations of the putative plant-related genes were also compared to determine any differential traits that selected the plant-associated micromonosporae from other environments (Supplementary Figure 4). PCA analysis also yielded three groups (referred to as *clusters*) with similar strain distribution to the COGs. The first cluster (C1) contained 30 members, with plant-associated strains representing 77%, soil/rhizosphere 16.6%, and marine/mangrove sediments accounting for 6.4%. The second cluster (C2) had 32 strains: soil/rhizosphere, 65.6%; mangrove/marine sediments, 12.5%; plant-related 6.3%; and other environments 15.6%. Cluster 3 (C3) was composed of twelve strains isolated from soil/rhizosphere (25%); mangrove/marine sediments (66.7%) and other environments, including the *Salinispora* strains (8.3%). Pearson chi-square test revealed a strong correlation between the strain clusters and their isolation source. A phylogenomic tree of the study strains, their habitat, and cluster assignment based on KEGG orthology is provided in Figure 3.

**FIGURE 4:**
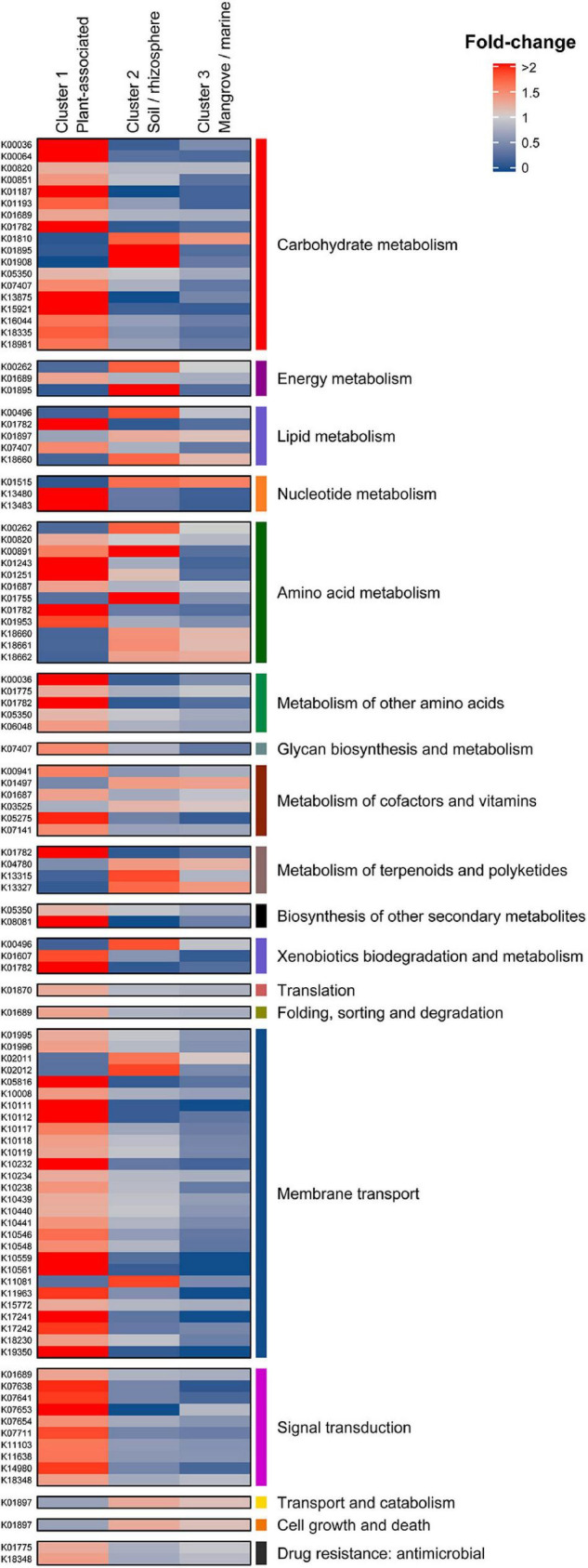
Differential KEGG annotations between Cluster 1 (Plant associated), Cluster 2 (Soil/rhizosphere), and Cluster 3 (Mangrove/marine). Annotations are grouped in their corresponding KEGG categories (first and last columns).

KEGG orthology (KO) revealed significant differences in the distribution of enriched gene functions within the three clusters. Cluster 1 (plant-associated) contained the highest number of overrepresented KEGG annotations with 105, followed by clusters 2 and 3 with 22 and 2 functions, respectively (Figure 4). Underrepresented functions were 20 (C1), 24 (C2), and 16 (C3). The full KEGG annotation list is found in Supplementary Table 5.

### Predictive Functional Signatures of Plant Associated *Micromonospora* Strains

Eighteen differential KO categories were identified as genomic signatures of *Micromonospora* plant-associated strains when compared to soil/rhizosphere and marine/mangrove habitats. Of these, the major categories were carbohydrate metabolism, membrane transport, amino acid metabolism and transport, signal transduction, metabolism of cofactors and vitamins, and nucleotide metabolism (Figure 4 and Supplementary Table 5).

Plant associated strains (C1) showed an important enrichment of genes related to carbohydrate metabolism, which decreased for the rhizosphere/soil related strains (C2) and were depleted in the mangrove/marine sediment isolates (C3) (except for glucose-6-phosphate isomerase). *B eta-*glucosidases that hydrolyze cellobiose released during the initial hydrolysis of cellulose (Medie et al., 2012), were found in as many as six copies in C1 strains. Also, genes coding for *L*-arabonate dehydratase (*ara*C) and arabinoxylan arabinofuranohydrolase (*xyn*D) were over-represented, with more than a two-fold difference with respect to the overall mean. In addition, *mal*Z, *sac*A, and *gal*A genes, coding for several sugar interconversions (e.g., raffinose, sucrose, and melibiose to glucose, galactose, and fructose) were found over-represented in the C1 isolates. These results are in line with previous results showing that the endophytic model strain *Micromonospora lupini* Lupac 08 contained a significant number of functional carbohydrate related genes, especially for degradation of plant-polysaccharides (Trujillo et al., 2014). Similar results were reported when plant-associated bacterial genomes were compared against those of non-plant environments, but phylogenetically related (Levy et al., 2018).

Transport systems are highly correlated to lifestyles and are essential for an organism to survive in a given environment (Ren and Paulsen, 2007). Several oligosaccharide transporters were found to be over-represented in C1 isolates (plant associated). ABC transporter genes for various sugars (e.g., *msm*X, K, E, F, and G) such as raffinose and melibiose were found highly over-enriched by more than two-fold change with respect to the overall mean. Part of the ribose ABC transport system coding genes (*rbs*A, B, and C) were also found with four to five gene copies per strain. These results correlate well with the carbohydrate metabolism category as many of the sugars released by the plant in the form of root exudates need to be introduced into the bacterial cell to serve as carbon sources. It was recently shown how several *Pseudomonas* strains responded to root exudates by inducing several transport systems that encoded a Major Facilitator Superfamily (MFS) transporter and an L-arabonate dehydratase, an important enzyme for the catabolism of arabinose (Mavrodi et al., 2021).

Amino acids secreted by the host plant can serve as carbon and nitrogen sources for plant-associated bacteria. In this category, genes related to the degradation of leucine, isoleucine, and valine were especially enriched. Interestingly, genes coding for branched-chain amino-acid transporters (*liv*) were also overrepresented in all strains in cluster 1 (plant-associated), with a mean of 10 genes per genome (*liv*G and *liv*F). Other enriched genes related to the metabolism of cysteine and methionine, tryptophan and lysine were found. A large proportion of genes encoding for proteins involved in amino acid transport and metabolism has been proposed as a key function in plant colonization (Cole et al., 2017). Similar results were also observed for good plant colonizers related to the sugarcane microbiome (de Souza et al., 2019).

Transduction systems are especially important for bacteria to respond to abrupt environmental changes. Seven KO categories related to signal transduction mechanisms were also identified as signatures of C1 strains. Five of these were related to two-component systems of the OmpR families. Several sensor histidine kinases were enriched by two-fold, including one representing an osmolarity sensor (EnvZ). In addition, a C4-dicarboxylate transport protein was found. The important role played by a new regulator from the OmpR family in the symbiosis of *Rhizobium etli* and *Phaseolus vulgaris* was recently reported (Rodríguez et al., 2020). Similarly, transcription regulators, related to biofilm formation, biosynthesis of antibiotics, response to osmotic stress and toxic chemicals, and pathogenicity were found enriched for bacteria colonizing plants (de Souza et al., 2019).

It is reported that vitamins can act as elicitors or priming agents to stimulate the plant defense mechanisms (Westman et al., 2019). Vitamins have also been reported to play an important role in root colonization (Lugtenberg et al., 2001; Babalola, 2010). Complete metabolic pathways for production of thiamine (B1), riboflavin (B2), niacin (B3), pantothenate (B5), pyridoxine (B6), biotin (B7), and folate (B9) were found in almost all *Micromonospora* genomes analyzed. The genes *thi*D, *ilv*D, and one coding for a pyridoxine 4-dehydrogenase (involved in B1, B6, and B5 biogenesis) were found significantly over-represented in the plant-associated cluster.

Urate is one of the main end products of rhizobial infected cells in legumes. It is transported to uninfected nodular cells where it is transformed into ureides that are transported in the xylem to the rest of the plant (Baral et al., 2016; Izaguirre-Mayoral et al., 2018). In this category, genes coding for xanthine dehydrogenases (*xdh*G and *yag*T), involved in the metabolization of urates, were over-represented in cluster 1 (>two-fold difference).

### Genomic Features of Clusters 2 and 3

Cluster 2 (soil/rhizosphere) shared an equal number of over and underrepresented functions and could be considered a transition cluster between 1 and 3. Cluster 3 (mangrove/marine sediments) was characterized by the low number of plant-related features, presenting only 18 differential features, 16 of them under-represented (Supplementary Table 5). Most of the under-represented features (fold < 0.5) were involved in carbon source metabolism and transport (*ara*A, *msm*FG, and several multiple sugar transport permease coding genes). Clearly, these results highly correlate with the origin of the strains.

### Effect of *Micromonospora* on *Arabidopsis*

**FIGURE 5:**
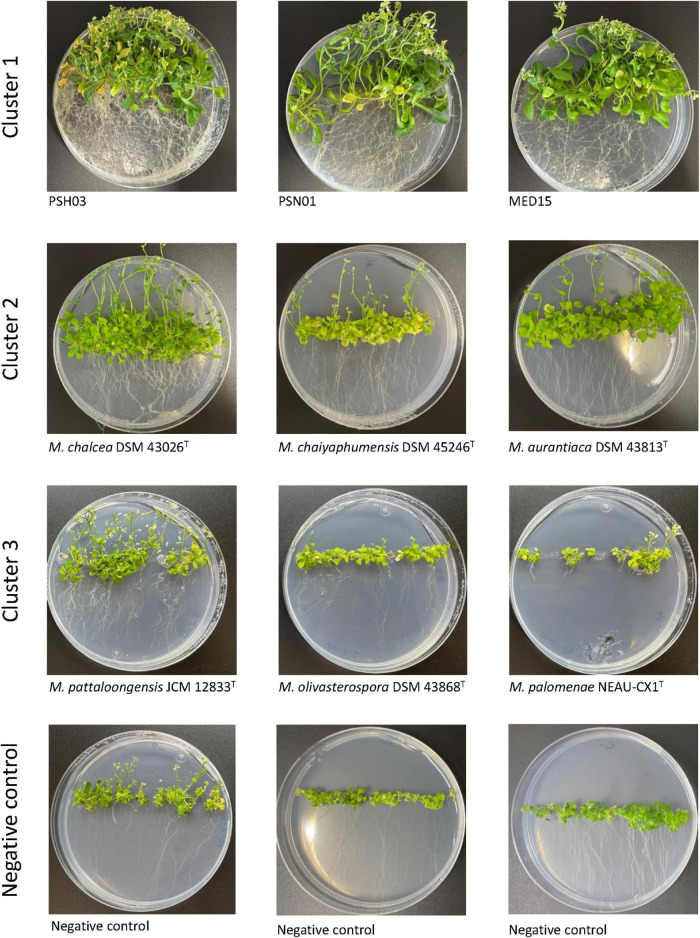
*Arabidopsis thaliana* plants after 4 weeks of growth and inoculated with strains from Cluster 1 (MED15, PSN01, and PSH03), Cluster 2 (*M. aurantiaca* DSM 43813*^T^*, *M. chaiyaphumensis* DSM 45246*^T^*, and *M. chalcea* DSM 43026*^T^*), and Cluster 3 (*M. pattaloongensis* JCM 12833*^T^*, *M. palomenae* DSM 102131*^T^*, and *M. olivasterospora* DSM 43868*^T^*).

**FIGURE 6:**
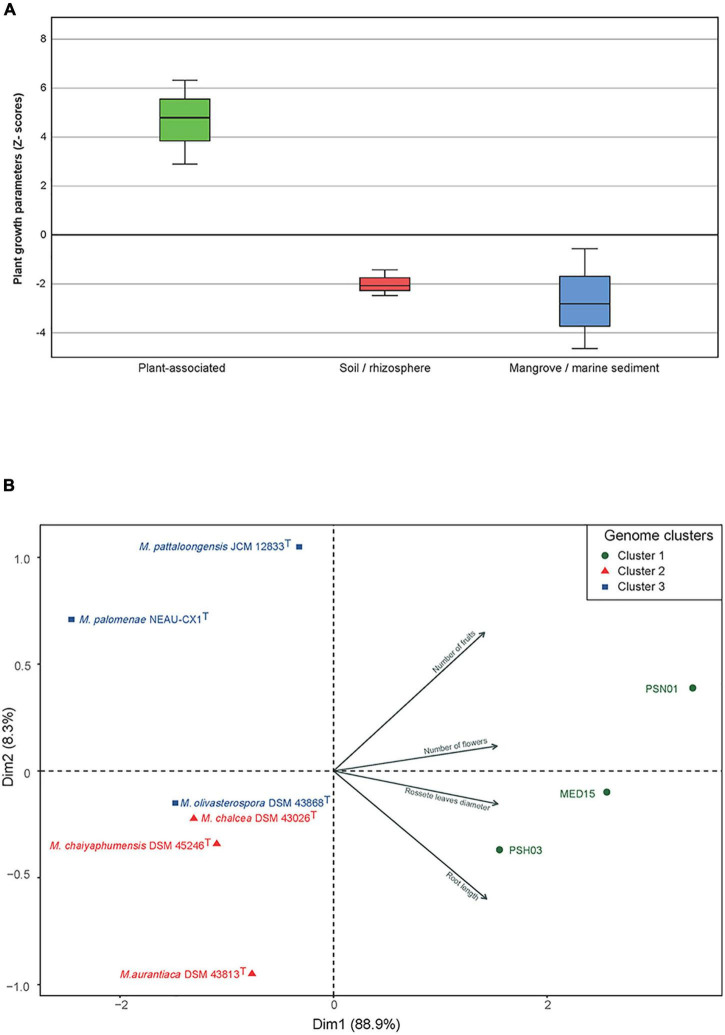
**(A)** Accumulated Z-scores of the growth parameters measured in *A. thaliana* after 4 weeks of growth and inoculated with *Micromonospora* strains selected from clusters C1, C2, and C3. **(B)** PCA distribution of the strains based on the plant growth parameters measured. Symbols: green circles, C1 (plant-associated); red triangles, C22 (soil/rhizosphere); blue squares, C3 (mangrove/marine sediments).

After 4 weeks, important growth differences were observed between the plants inoculated with selected strains from the three different environments. Those treated with the plant-associated isolates (cluster 1) showed the best growth and development, followed by the plants inoculated with strains from soil/rhizosphere (cluster 2). The least growth was obtained for the plants inoculated with the strains from mangrove/sediment (cluster 3) where growth was similar to the control plants, except for the ones treated with *M. pattaloongensis* JCM 12833*^T^* (Figure 5). Overall Z-scores of the 360 plants inoculated with the different isolates showed that strains PSH03, PSN01, and MED15 (plant-associated) had the highest effect on the *Arabidopsis* plants (Figure 6A). The number of flowers and fruits, root length, and rosette leaf diameter values highly correlated in the PCA analysis with these strains (Figure 6B). Interestingly, all strains used for plant inoculations shared common markers identified as plant growth promotion characteristics.

## Discussion

*Micromonospora*, a common bacterium in soils and aquatic habitats was reported more than 10 years ago, as part of the legume nitrogen fixing nodule microbiome (Trujillo et al., 2010; Carro et al., 2012). This actinobacterium has gained interest, given its potential use in combination with rhizobia to enhance legume growth and nitrogen fixation (Martínez-Hidalgo and Hirsch, 2017).

The number of *Micromonospora* genomes sequenced has increased in recent years facilitating comparative genomic analyses in search for plant-growth promotion traits (Trujillo et al., 2014; Carro et al., 2018). Despite this increase, representative genomes of strains isolated from plant tissues (e.g., nodules, roots, etc.) is still low when compared to the soil environment. In this work we sequenced 17 new genomes from *Micromonospora* strains that were previously isolated from several legumes (Riesco et al., 2018; Benito et al., 2022). A working database containing 74 *Micromonospora* genomes with an almost equal number of soil- and plant-related representatives was used as the basis of this work. Using a novel comparative genomic approach that combined a bacterial plant-related database (Levy et al., 2018) and the proteome of *Micromonospora* host plants, we determined a set of genomic features that suggest a strong relation to plants.

It was recently suggested that bacterial association to plants is partially reflected in the size of the bacterial genome (larger) as compared to those which are not associated (smaller) (Levy et al., 2018). In this study, no significant correlation between genome size and environment was found. Furthermore, genome size in the two main isolation habitats (soil and plants) was very similar (7.1 ± 0.4 Mbp). As expected, the genome sizes of the *Micromonospora* and *Salinispora* strains varied greatly, with a mean difference of 1.5 Mb. While these two microorganisms are phylogenetically closely related, important differences can be found at the genomic level. *Salinispora* is a marine obligate bacterium, and its reduced genome strongly suggests an adaptation to this environment. *Micromonospora* on the other hand, appears to have evolved to adapt to multiple niches which could be translated in larger genomes to accommodate different life styles (Trujillo et al., 2014).

To select for plant-associated bacteria and especially those that provide a benefit to the host, PGP traits are commonly used as selective markers. In the present study, several genomic characteristics commonly related to plant growth promoting bacteria were initially included in the pool of 69,046 genes and tagged as plant-related genes (e.g., siderophores, phytohormones, etc.), however, most of these traits were not part of the final list of genomic signatures that differentiated between the strains and their environments. In fact, many strains included in this work were previously screened for common PGP traits and most yielded positive activities for production of siderophores, indol-acetic acid, and ACC deaminase, irrespective of their origin (Ortúzar, 2017). Thus, the presence of PGP traits does not appear to be reliable for the selection of strains that will successfully colonize a plant and interact with it (Finkel et al., 2017; Cai et al., 2018; de Souza et al., 2019).

Root exudates play a key role in the selection of bacterial communities that colonize a plant and serve as mediators in the establishment of both positive and negative interactions in the rhizosphere (Badri and Vivanco, 2009; Chaparro et al., 2014). Exudates include sugars, amino acids, fatty acids, sterols, phenolics, and organic acids that serve as carbon and energy sources for the surrounding bacteria, initiating a cross-talk which may result in successful root colonization of the host (Nguyen et al., 2003; Badri and Vivanco, 2009; Mavrodi et al., 2021). The genomic signatures defined for the plant-associated micromonosporae were especially rich in genes coding for carbohydrate metabolism, membrane transport, amino acid metabolism, and signal transduction, strongly suggesting that these features are especially important in establishing communication and successful root colonization. Transcriptomic analysis of several *Micromonospora* strains in contact with lupin root exudates demonstrated that the *msm* and *rsb* systems found enriched in this work, were up regulated (Benito, 2020). Similarly, a recent study showed how the genomes of a bacterial community of “robust colonizers” in maize were enriched in similar metabolic functions (de Souza et al., 2019). Interestingly, these authors also reported that PGP features were not determinant for a successful colonization (de Souza et al., 2019). Both results are in line with those obtained in the present work.

*In vitro* production of plant cell wall hydrolytic enzymes (e.g., cellulases, xylanases, amylases, etc.) was previously demonstrated in *Micromonospora* strains isolated from nitrogen fixing nodules (Trujillo et al., 2014; Benito et al., 2022). When some of these strains were exposed to the lupin root exudates, several α*-* and β-glucosidases were found overexpressed (four to ten-fold) (Benito, 2020). These enzymes are known to play a key role in bacterial root colonization and tissue penetration (Reinhold-Hurek et al., 2006; Liu et al., 2017; Compant et al., 2021). In addition, cellulases are not restricted to cellulose hydrolysis but could be involved in other biological functions (Medie et al., 2012); they have also been shown to be essential for root infection in rhizobia (Robledo et al., 2012). Furthermore, plant hydrolytic enzymes were also found highly represented in metagenomic samples of endophytic bacterial communities residing inside rice roots (Sessitsch et al., 2012). Overall, carbohydrate metabolism and its corresponding transports are clearly a main feature of plant-associated bacteria.

Amino acids are exudated by many plants and can be used as carbon and nitrogen sources by bacteria surrounding the rhizosphere (Badri and Vivanco, 2009). Within these molecules, branched-chain amino acids (LIV) are recognized as important factors in the bacteroid-legume relationship as they serve as nitrogen sources for the bacteroid (Prell et al., 2009a,b). LIV transporters are essential to help LIVs move across the symbiosome membrane to make nitrogen available to the bacteroids inside (Prell et al., 2009b). In this study, LIV transporters were found overrepresented with an average of 10 copies in the genomes of strains associated with an endophytic lifestyle (cluster 1). It was previously reported that *Micromonospora* increases nutrition efficiency in *Medicago* (Trujillo et al., 2014; Martínez-Hidalgo et al., 2015). *Micromonospora* could act as a backup system for the provision of LIV transporters to secure good bacteroid development and subsequently efficient nitrogen fixation. LIV transporters were also enriched in the bacterial community of root colonizers in maize, strongly suggesting that amino acid metabolism and transport play a key role in plant-microbe interactions and is not restricted to the rhizobium-legume symbiosis (de Souza et al., 2019).

Glutamine and arginine together with ureides are end products in nitrogen fixing nodules. These molecules are transported through the xylem to other plant organs (e.g., leaves) and serve as sources of N (Baral et al., 2016; Izaguirre-Mayoral et al., 2018). In the case of ureides these are the final products in determinate nodules, while amino acids are found in plant species with indeterminate ones (e.g., lupin). Interestingly, plant-associated micromonosporae (cluster 1) have been found in both types of nodules (Trujillo et al., 2010; Carro et al., 2012). Purine metabolism involving plants and their associated bacteria is very complex and includes various metabolic pathways (Izaguirre-Mayoral et al., 2018). Apart from rhizobia and nitrogen fixation in legumes, it is not clear how other bacteria (e.g., *Micromonospora*) participate.

Recent studies have demonstrated that vitamins can be used to prime plant defenses against pathogens and abiotic stress (Boubakri et al., 2016; Westman et al., 2019). Specifically, thiamine has been shown to activate systemic acquired resistance (SAR) in plants against pathogens (Ahn et al., 2005). B-complex vitamins which act as coenzymes in several metabolic processes such as glycolysis, Krebs cycle, and nucleic acid synthesis among others, are produced by plants and microbes, including bacteria that are present in the microbiome of a plant and could, in turn, supply vitamins to enhance plant resistance.

The bacterial transcripts of three strains from C1 (*M. cremea* CR30*^T^*, *M. lupini* Lupac 08, and *M. saelicesensis* Lupac 09*^T^*) were obtained when grown in contact with lupin root exudates. Various genes involved in the transport of sugars and aminoacids (*rbs* and *liv*), multiple sugar transporters (*msm* and ABC-MS), synthesis of vitamins (*ilv*D, *coa*X and *moc*A), and carbohydrate hydrolysis (e.g., *gal*A, *bgl*B, and *ara*C) were found overexpressed (Benito, 2020). These results are interesting as they coincide with some of the metabolic functions found in this work. However, it is necessary to fully validate the genomic signatures with additional plant assays that include gene expression analyses upon exposure of the bacterium to the host plant, not only to the root exudates. In this line, *in-planta* assays in combination with transcriptomic analyses are underway.

Important differences in plant phenotype were found when *Arabidopsis* plants were inoculated with *Micromonospora* strains selected from the three different environment clusters defined by the genomic traits identified. All strains had previously been screened for PGP characteristics that included among others, siderophore, IAA, and AC deaminase production, yielding a positive reaction. These findings strongly suggest that PGP markers alone, are not good indicators for the selection of bacterial strains to develop a desired phenotype, especially to increase crop production or the recovery of ecosystems.

## Conclusion

The genomic features defined in this work, using a new bioinformatic pipeline confirm and expand those previously identified in the bacterial adaptation process to plants. Other studies have shown that several of these genomic markers are also present in phylogenetically diverse bacterial taxa that interact with non-leguminous plants. Highly related genomes of *Micromonospora* strains isolated from diverse habitats, were separated in three clusters and their genomic differences (genomic signatures) could be used to select for strains with the highest probability to successfully colonize and interact with a host plant. Many of the genes commonly identified as PGP did not have any weight as differential characteristics in the new database, therefore their presence is not necessarily a good indication to establish a successful interaction with the host plant. These genetic markers could be considered in microbiome engineering when *Micromonospora* strains are included as part of a consortium aiming to create predictable plant phenotypes.

## Data Availability Statement

The datasets presented in this study can be found in online repositories. The names of the repository/repositories and accession number(s) can be found in the article/Supplementary Material.

## Author Contributions

RR: investigation, methodology, software development, data analysis, and writing. MO: investigation, data analysis, and writing. JF-Á: conceptualization, supervision, funding resources, and writing. MT: conceptualization, methodology, funding resources, project supervision, and writing. All authors contributed to the article and approved the submitted version.

## Conflict of Interest

The authors declare that the research was conducted in the absence of any commercial or financial relationships that could be construed as a potential conflict of interest.

## Publisher’s Note

All claims expressed in this article are solely those of the authors and do not necessarily represent those of their affiliated organizations, or those of the publisher, the editors and the reviewers. Any product that may be evaluated in this article, or claim that may be made by its manufacturer, is not guaranteed or endorsed by the publisher.

## Footnotes

1
https://www.ezbiocloud.net/tools/ubcg

2
https://github.com/mgoeker/table2itol
