# Supplementary figures and images for: Deciphering Genomes: Genetic Signatures of Plant-Associated Micromonospora
Source: Front Plant Sci. 2022 Mar 25;13:872356. doi: 10.3389/fpls.2022.872356 (PMC8990736; doi:10.3389/fpls.2022.872356)

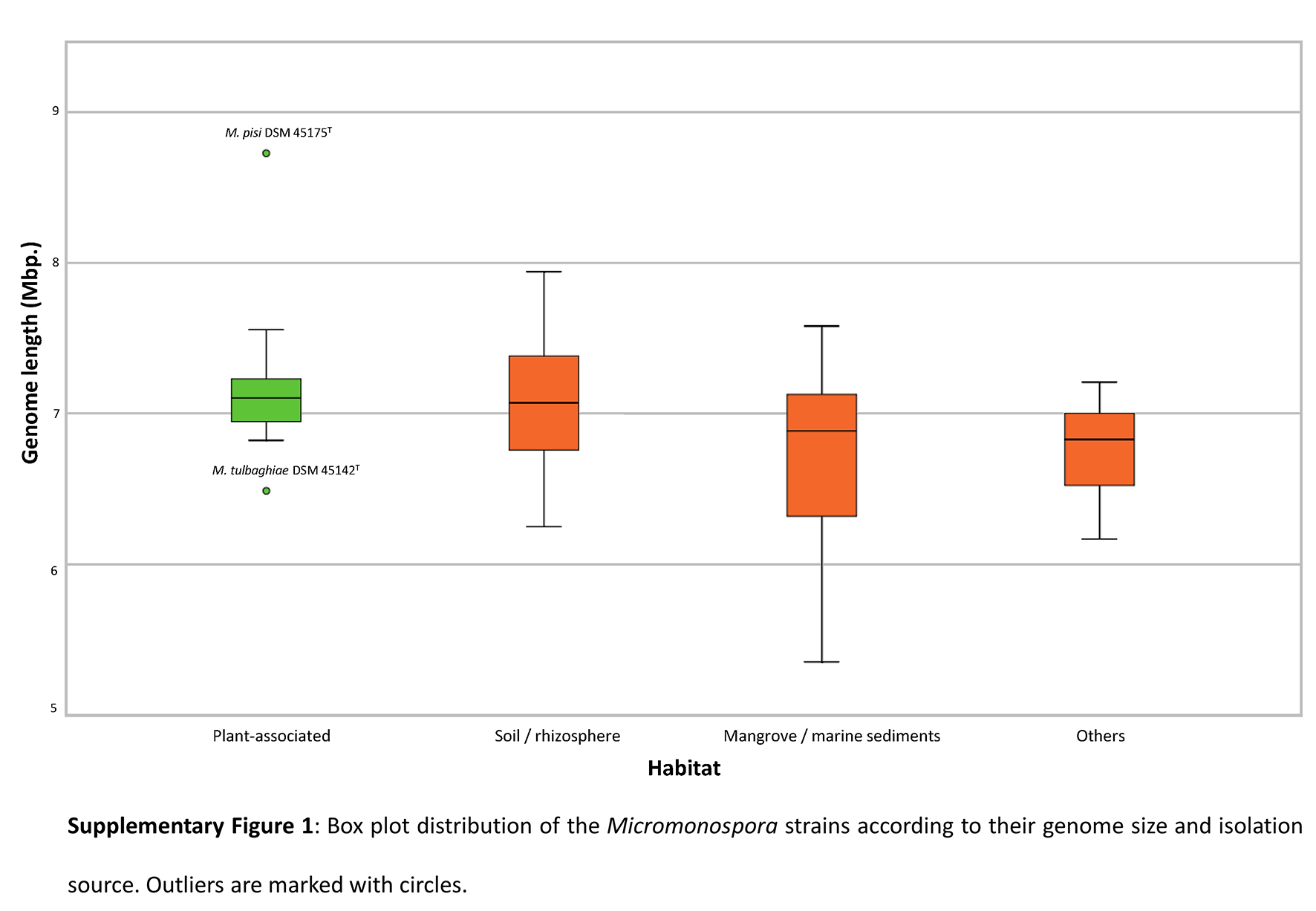

Supplement: Supplementary file 1 [file Image_1.TIF]

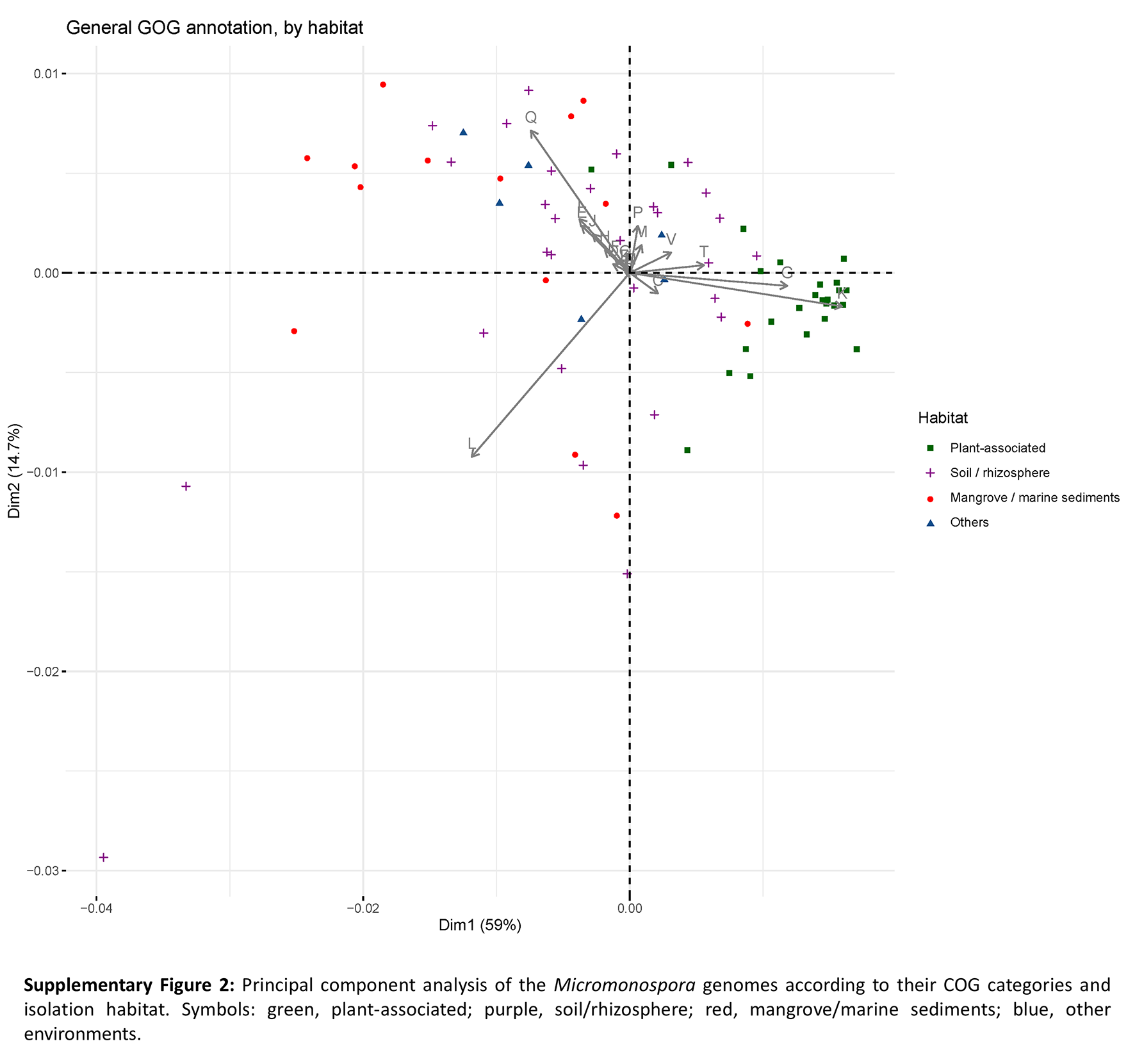

Supplement: Supplementary file 2 [file Image_2.TIF]

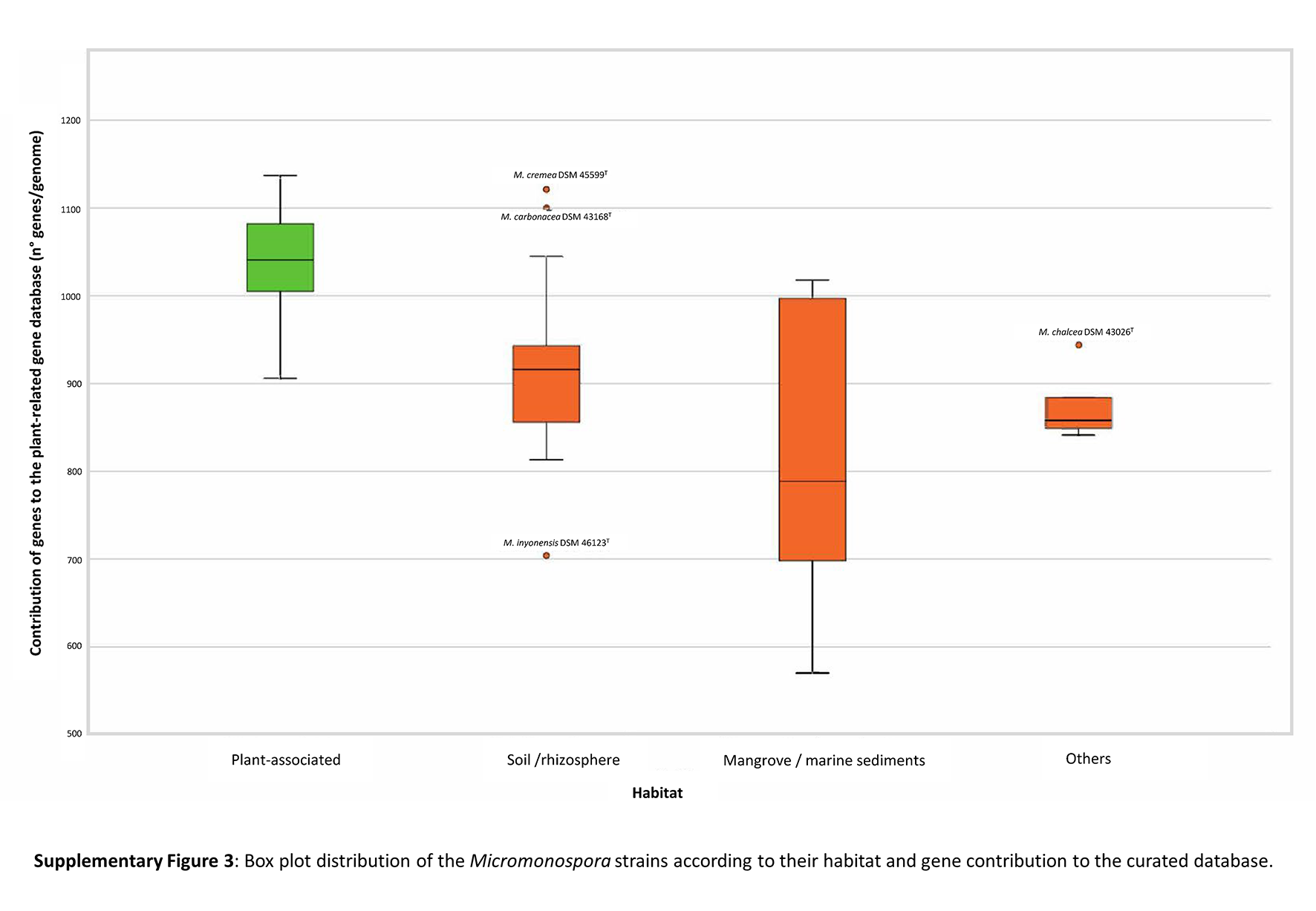

Supplement: Supplementary file 3 [file Image_3.TIF]

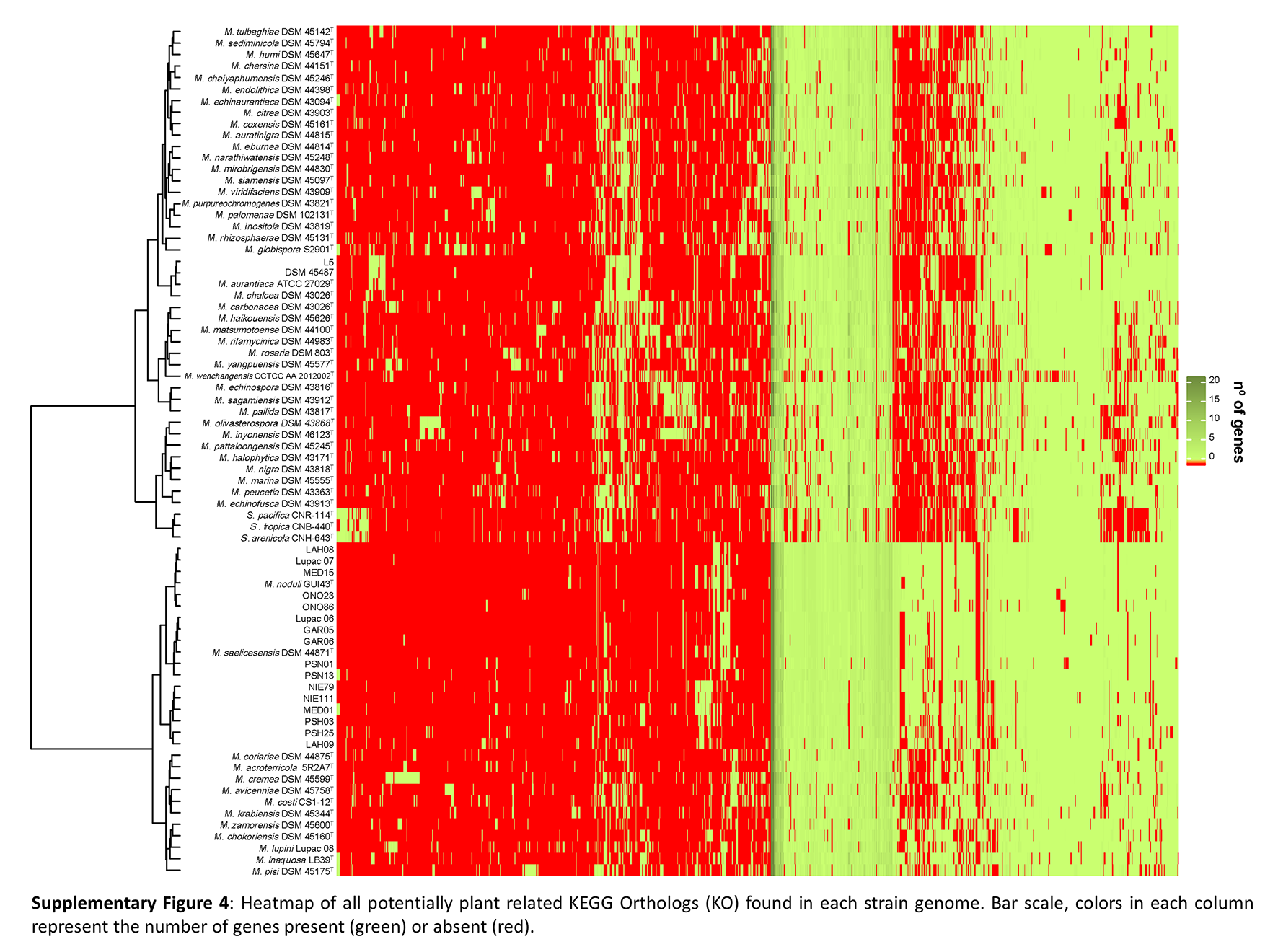

Supplement: Supplementary file 4 [file Image_4.TIF]
