# Supplementary material for: Deciphering Genomes: Genetic Signatures of Plant-Associated Micromonospora
Source: Front Plant Sci. 2022 Mar 25;13:872356. doi: 10.3389/fpls.2022.872356 (PMC8990736; doi:10.3389/fpls.2022.872356)
Supplement: Supplementary file 5 [file Table_1.DOCX]

##### **Supplementary Table 1**: General genome characteristics of the sequenced genomes. CRISPR number only represent only elements marked as “confirmed CRISPR” in the CRISPRfinder tool, all questionable CRISPR have been omitted.

| Strain | Genome length (Mb) | G+C ratio (mol%) | CDS | rRNA | tRNA | Contigs | CRISPR | Depth | N50 |
| --- | --- | --- | --- | --- | --- | --- | --- | --- | --- |
| GAR05 | 7.1 | 71.2 | 6526 | 6 | 68 | 75 | 0 | 176 | 162179 |
| GAR06 | 7.0 | 71.2 | 6410 | 8 | 67 | 60 | 4 | 275 | 180642 |
| *M. noduli* GUI43^T^ | 7.2 | 70.9 | 6539 | 3 | 57 | 225 | 0 | 784 | 105196 |
| LAH08 | 7.3 | 71.1 | 6627 | 4 | 56 | 62 | 3 | 170 | 278302 |
| LAH09 | 6.9 | 71.6 | 6182 | 4 | 50 | 133 | 1 | 270 | 102519 |
| Lupac 06 | 7.1 | 71.2 | 6495 | 4 | 65 | 59 | 1 | 235 | 191999 |
| Lupac 07 | 7.1 | 71.1 | 6500 | 4 | 51 | 56 | 2 | 216 | 240674 |
| MED01 | 7.6 | 70.8 | 7060 | 4 | 53 | 63 | 3 | 246 | 232044 |
| MED15 | 7.2 | 71.1 | 6554 | 4 | 56 | 43 | 1 | 191 | 354266 |
| NIE111 | 6.9 | 71.1 | 6298 | 3 | 52 | 71 | 2 | 155 | 232317 |
| NIE79 | 7.2 | 71.1 | 6566 | 5 | 84 | 82 | 3 | 155 | 168635 |
| ONO23 | 7.2 | 71.0 | 6565 | 5 | 56 | 135 | 2 | 315 | 99720 |
| ONO86 | 7.1 | 70.9 | 6591 | 4 | 55 | 407 | 2 | 322 | 29811 |
| PSH03 | 7.0 | 71.0 | 6395 | 4 | 54 | 45 | 2 | 342 | 295666 |
| PSH25 | 6.8 | 71.2 | 6395 | 5 | 45 | 586 | 2 | 303 | 19082 |
| PSN01 | 6.9 | 71.1 | 6458 | 4 | 67 | 456 | 4 | 361 | 26003 |
| PSN13 | 7.4 | 71.1 | 6823 | 3 | 64 | 41 | 1 | 227 | 485329 |
