# Supplementary material for: Deciphering Genomes: Genetic Signatures of Plant-Associated Micromonospora
Source: Front Plant Sci. 2022 Mar 25;13:872356. doi: 10.3389/fpls.2022.872356 (PMC8990736; doi:10.3389/fpls.2022.872356)
Supplement: Supplementary file 6 [file Table_2.DOCX]

**Supplementary Table 2**: Sequence accession numbers and isolation source of the genomes used in this work.

| Designation | Isolation source | Accession number | Genome size (Mb) |
| --- | --- | --- | --- |
| *M. acroterricola* 5R2A7^T^ | Desert soil | QGKR00000000 | 6.5 |
| *M. aurantiaca* ATCC 27029^T^ | Soil | NC_014391 | 7.5 |
| *M. aurantiaca* DSM 45487 | Humic Soil | FMHX00000000 | 7.4 |
| *M. aurantiaca* L5 | Nodules of *Casuarina* *equisetifolia* | NC_014815 | 7.0 |
| *M. auratinigra* DSM 44815^T^ | Peat swamp forest soil | LT594323 | 6.8 |
| *M. avicenniae* DSM 45758^T^ | Root of *Avicennia marina* | FTNF00000000 | 6.8 |
| *M. carbonacea* DSM 43168^T^ | Soil | FMCT00000000 | 7.9 |
| *M. chaiyaphumensis* DSM 45246^T^ | Mountain Soil | FMCS00000000 | 6.7 |
| *M. chalcea* DSM 43026^T^ | Air | MAGP00000000 | 7.0 |
| *M. chersina* DSM 44151^T^ | Soil | FMIB00000000 | 6.7 |
| *M. chokoriensis* DSM 45160^T^ | Sandy soil | LT607409 | 6.9 |
| *M. citrea* DSM 43903^T^ | Lake mud | FMHZ00000000 | 7.2 |
| *M. coriariae* DSM 44875^T^ | Nodules of *Coriaria myrtifolia* | LT607412 | 6.9 |
| *M. costi* CS1-12^T^ | Leaves of *Costus speciosus* | RBAN00000000 | 7.2 |
| *M. coxensis* DSM 45161^T^ | Sandy soil | LT607753 | 6.8 |
| *M. cremea* DSM 45599^T^ | Rhizosphere of *Pisum sativum* | FSQT00000000 | 7.8 |
| *M. eburnea* DSM 44814^T^ | Peat swamp forest soil | FMHY00000000 | 7.2 |
| *M. echinaurantiaca* DSM 43094^T^ | Soil | LT607750 | 7.2 |
| *M. echinofusca* DSM 43913^T^ | Excrement of chukar | LT607733 | 7.0 |
| *M. echinospora* DSM 43816^T^ | Soil | LT607413 | 7.7 |
| *M. endolithica* DSM 44398^T^ | Sandstone rock | VLLO00000000 | 7.0 |
| *M. globispora* S2901^T^ | Marine sediment | QGGF00000000 | 6.7 |
| *M. haikouensis* DSM 45626^T^ | Mangrove soil | FMCW00000000 | 7.6 |
| *M. halophytica* DSM 43171^T^ | Salt pool | FMDN00000000 | 6.3 |
| *M. humi* DSM 45647^T^ | Peat swamp forest soil | FMDM00000000 | 6.7 |
| *M. inaquosa* LB39^T^ | Desert soil | QGSZ00000000 | 7.7 |
| *M. inositola* DSM 43819^T^ | Forest soil | LT607754 | 6.7 |
| *M. inyonensis* DSM 46123^T^ | Soil | FMHU00000000 | 6.9 |
| *M. krabiensis* DSM 45344^T^ | Marine soil | LT598496 | 7.1 |
| *M. lupini* Lupac 08 | Nodules of *Lupinus angustifolius* | CAIE00000000 | 7.3 |
| *M. marina* DSM 45555^T^ | Sea sand | FMCV00000000 | 6.1 |
| *M. matsumotoense* DSM 44100^T^ | Woodland Soil | FMCU00000000 | 7.8 |
| *M. mirobrigensis* DSM 44830^T^ | Freshwater pond | FMCX00000000 | 6.2 |
| *M. narathiwatensis* DSM 45248^T^ | Peat swamp forest soil | LT594324 | 6.6 |
| *M. nigra* DSM 43818^T^ | Salt pool | FMHT00000000 | 6.4 |
| *M. noduli* GUI43^T^ | Nodules of *Pisum sativum* | PYAK00000000 | 7.2 |
| *M. noduli* LAH08 | Leaves of *Lupinus* sp. | PYAA00000000 | 7.2 |
| *M. noduli* Lupac 07 | Nodules of *Lupinus* *angustifolius* | PYAB00000000 | 7.1 |
| *M. noduli* MED15 | Nodules of *Medicago* sp. | PYAC00000000 | 7.2 |
| *M. noduli* ONO23 | Nodules of *Ononis* sp. | PYAD00000000 | 7.2 |
| *M. noduli* ONO86 | Nodules of *Ononis* sp. | PYAE00000000 | 7.1 |
| *M. olivasterospora* DSM 43868^T^ | Soil | VLKE00000000 | 7.1 |
| *M. pallida* DSM 43817^T^ | Soil | FMHW00000000 | 7.8 |
| *M. palomenae* DSM 102131^T^ | Nymphs of stinkbug (*Palomena viridissima*) | VIXA00000000 | 6.7 |
| *M. pattaloongensis* DSM 45245^T^ | Mangrove forest soil | FNPH00000000 | 5.3 |
| *M. peucetia* DSM 43363^T^ | Soil | FMIC00000000 | 7.4 |
| *M. pisi* DSM 45175^T^ | Nodules of *Pisum sativum* | RBKT00000000 | 8.7 |
| *M. purpureochromogenes* DSM 43821^T^ | Adobe soil | LT607410 | 6.9 |
| *M. rhizosphaerae* DSM 45131^T^ | Mangrove rhizospheric soil | FMHV00000000 | 7.2 |
| *M. rifamycinica* DSM 44983^T^ | Mangrove sediment | LT607752 | 7.0 |
| *M. rosaria* DSM 803^T^ | Soil | LRQV00000000 | 7.4 |
| *M. saelicesensis* DSM 44871^T^ | Nodules of *Lupinus* *angustifolius* | FMCR00000000 | 7.1 |
| *M. saelicesensis* GAR05 | Nodules of *Cicer* sp. | PXXW00000000 | 7.1 |
| *M. saelicesensis* GAR06 | Nodules of *Cicer* sp. | PYAH00000000 | 7.0 |
| *M. saelicesensis* Lupac 06 | Nodules of *Lupinus* *angustifolius* | PYAJ00000000 | 7.1 |
| *M. saelicesensis* PSN01 | Nodules of *Pisum* sp. | PYAI00000000 | 6.9 |
| *M. saelicesensis* PSN13 | Nodules of *Pisum* sp. | PYAG00000000 | 7.4 |
| *M. sagamiensis* DSM 43912^T^ | Soil | VLLP00000000 | 6.9 |
| *M. sediminicola* DSM 45794^T^ | Marine sediment | FLRH00000000 | 6.9 |
| *M. siamensis* DSM 45097^T^ | Peat swamp forest soil | LT607751 | 6.2 |
| *M. tulbaghiae* DSM 45142^T^ | Leaves of *Tulbaghia violacea* | FMCQ00000000 | 6.5 |
| *M. viridifaciens* DSM 43909^T^ | Soil | LT607411 | 7.1 |
| *M. wenchangensis* CCTCC AA 2012002^T^ | Mangrove soil | MZMV00000000 | 7.5 |
| *M. yangpuensis* DSM 45577^T^ | Sea sponge | FMIA00000000 | 6.5 |
| *M. zamorensis* DSM 45600^T^ | Rhizosphere of *Pisum* *sativum* | LT607755 | 7.1 |
| *Micromonospora* sp. LAH09 | Leaves of *Lupinus* sp. | JAKKFI000000000 | 6.9 |
| *Micromonospora* sp. MED01 | Nodules of *Medicago* sp. | JAKKFH000000000 | 7.6 |
| *Micromonospora* sp. NIE111 | Nodules of *Trifolium* sp. | JAKKFE000000000 | 6.9 |
| *Micromonospora* sp. NIE79 | Nodules of *Trifolium* sp. | JAKKFD000000000 | 7.2 |
| *Micromonospora* sp. PSH03 | Leaves of *Pisum* sp. | JAKKFG000000000 | 7.0 |
| *Micromonospora* sp. PSH25 | Leaves of *Pisum* sp. | JAKKFF000000000 | 6.8 |
| *S. arenicola* CNH-643^T^ | Marine sediment | jgi_2561511037 | 5.6 |
| *S. pacifica* CNR-114^T^ | Marine sediment | AZWO00000000 | 5.9 |
| *S. tropica* CNB-440^T^ | Marine sediment | NC_009380 | 5.2 |
