# Supplementary material for: Deciphering Genomes: Genetic Signatures of Plant-Associated Micromonospora
Source: Front Plant Sci. 2022 Mar 25;13:872356. doi: 10.3389/fpls.2022.872356 (PMC8990736; doi:10.3389/fpls.2022.872356)
Supplement: Supplementary file 8 [file Table_4.DOCX]

**Supplementary Table 4**: Number of genes included in the plant-related gene database. All genes in the core genome were deleted from the final database and are not included in the table. Plant-related genes (PR genes); Plant-resembling bacterial genes (PRB genes), Putative plant-related genes (PPR).

| Strain | PR unique genes | PRB unique genes | PR and PRB shared genes | Total PPR genes in database |
| --- | --- | --- | --- | --- |
| *M. acroterricola* 5R2A7^T^ | 668 | 186 | 89 | 943 |
| *M. aurantiaca* ATCC 27029^T^ | 598 | 215 | 88 | 901 |
| *M. aurantiaca* DSM45487 | 601 | 218 | 90 | 909 |
| *M. aurantiaca* L5 | 597 | 218 | 95 | 910 |
| *M. auratinigra* DSM 44815^T^ | 603 | 196 | 85 | 884 |
| *M. avicenniae* DSM 45758^T^ | 660 | 207 | 102 | 969 |
| *M. carbonacea* DSM 43168^T^ | 676 | 286 | 138 | 1100 |
| *M. chaiyaphumensis* DSM 45246^T^ | 624 | 211 | 86 | 921 |
| *M. chalcea* DSM 43026^T^ | 609 | 239 | 96 | 944 |
| *M. chersina* DSM 44151^T^ | 641 | 204 | 92 | 937 |
| *M. chokoriensis* DSM 45160^T^ | 645 | 195 | 95 | 935 |
| *M. citrea* DSM 43903^T^ | 572 | 187 | 82 | 841 |
| *M. coriariae* DSM 44875^T^ | 687 | 211 | 96 | 994 |
| *M. costi* CS1-12^T^ | 710 | 200 | 95 | 1005 |
| *M. coxensis* DSM 45161^T^ | 593 | 193 | 86 | 872 |
| *M. cremea* DSM 45599^T^ | 713 | 281 | 127 | 1121 |
| *M. eburnea* DSM 44814^T^ | 566 | 215 | 73 | 854 |
| *M. echinaurantiaca* DSM 43094^T^ | 651 | 206 | 86 | 943 |
| *M. echinofusca* DSM 43913^T^ | 572 | 197 | 81 | 850 |
| *M. echinospora* DSM 43816^T^ | 678 | 229 | 103 | 1010 |
| *M. endolithica* DSM 44398^T^ | 617 | 214 | 85 | 916 |
| *M. globispora* S2901^T^ | 635 | 209 | 122 | 966 |
| *M. haikouensis* DSM 45626T | 712 | 204 | 81 | 997 |
| *M. halophytica* DSM 43171^T^ | 459 | 185 | 83 | 727 |
| *M. humi* DSM 45647^T^ | 642 | 215 | 88 | 945 |
| *M. inaquosa* LB39^T^ | 722 | 219 | 104 | 1045 |
| *M. inositola* DSM 43819^T^ | 644 | 205 | 89 | 938 |
| *M. inyonensis* DSM 46123^T^ | 432 | 198 | 74 | 704 |
| *M. krabiensis* DSM 45344^T^ | 732 | 201 | 85 | 1018 |
| *M. lupini* Lupac 08 | 690 | 284 | 126 | 1100 |
| *M. marina* DSM 45555^T^ | 444 | 186 | 68 | 698 |
| *M. matsumotoense* DSM 44100^T^ | 674 | 218 | 86 | 978 |
| *M. mirobrigensis* DSM 44830^T^ | 600 | 193 | 91 | 884 |
| *M. narathiwatensis* DSM 45248^T^ | 570 | 196 | 75 | 841 |
| *M. nigra* DSM 43818^T^ | 478 | 167 | 72 | 717 |
| *M. noduli* GUI43^T^ | 725 | 243 | 114 | 1082 |
| *M. noduli* LAH08 | 741 | 224 | 104 | 1069 |
| *M. noduli* Lupac 07 | 735 | 220 | 103 | 1058 |
| *M. noduli* MED15 | 741 | 242 | 111 | 1094 |
| *M. noduli* ONO23 | 755 | 219 | 103 | 1077 |
| *M. noduli* ONO86 | 712 | 209 | 105 | 1026 |
| *M. olivasterospora* DSM 43868^T^ | 501 | 229 | 83 | 813 |
| *M. pallida* DSM 43817^T^ | 572 | 208 | 90 | 870 |
| *M. palomenae* DSM 102131^T^ | 573 | 195 | 81 | 849 |
| *M. pattaloongensis* DSM 45245^T^ | 506 | 172 | 64 | 742 |
| *M. peucetia* DSM 43363^T^ | 551 | 219 | 93 | 863 |
| *M. pisi* DSM 45175^T^ | 805 | 227 | 105 | 1137 |
| *M. purpureochromogenes* DSM 43821^T^ | 572 | 201 | 83 | 856 |
| *M. rhizosphaerae* DSM 45131^T^ | 667 | 236 | 96 | 999 |
| *M. rifamycinica* DSM 44983^T^ | 569 | 189 | 77 | 835 |
| *M. rosaria* DSM 803^T^ | 658 | 208 | 90 | 956 |
| *M. saelicesensis* DSM 44871^T^ | 716 | 235 | 105 | 1056 |
| *M. saelicesensis* GAR05 | 724 | 214 | 100 | 1038 |
| *M. saelicesensis* GAR06 | 716 | 220 | 105 | 1041 |
| *M. saelicesensis* Lupac 06 | 719 | 221 | 102 | 1042 |
| *M. saelicesensis* PSN01 | 706 | 205 | 97 | 1008 |
| *M. saelicesensis* PSN13 | 768 | 225 | 106 | 1099 |
| *M. sagamiensis* DSM 43912^T^ | 579 | 194 | 83 | 856 |
| *M. sediminicola* DSM 45794^T^ | 624 | 206 | 82 | 912 |
| *M. siamensis* DSM 45097^T^ | 537 | 196 | 83 | 816 |
| *M. tulbaghiae* DSM 45142^T^ | 607 | 211 | 88 | 906 |
| *M. viridifaciens* DSM 43909^T^ | 562 | 215 | 76 | 853 |
| *M. wenchangensis* CCTCC AA 2012002^T^ | 595 | 295 | 122 | 1012 |
| *M. yangpuensis* DSM 45577^T^ | 516 | 249 | 101 | 866 |
| *M. zamorensis* DSM 45600^T^ | 628 | 211 | 92 | 931 |
| *Micromonospora* sp. LAH09 | 682 | 201 | 89 | 972 |
| *Micromonospora* sp. MED01 | 747 | 239 | 105 | 1091 |
| *Micromonospora* sp. NIE111 | 685 | 238 | 99 | 1022 |
| *Micromonospora* sp. NIE79 | 744 | 241 | 100 | 1085 |
| *Micromonospora* sp. PSH03 | 691 | 211 | 99 | 1001 |
| *Micromonospora* sp. PSH25 | 707 | 213 | 98 | 1018 |
| *S. arenicola* CNH-643^T^ | 359 | 194 | 76 | 629 |
| *S. pacifica* CNR-114^T^ | 327 | 184 | 68 | 579 |
| *S. tropica* CNB-440^T^ | 325 | 181 | 64 | 570 |
